# Ectopic Expression of Human *DPPA2* Gene in ESCC Cell Line Using Retroviral System

**Published:** 2018

**Authors:** Maryam Khaleghizadeh, Mohammad Mahdi Forghanifard, Abolfazl Rad, Moein Farshchian, Zahra Hejazi, Mehran Gholamin, Bahram Memar, Mohammad Reza Abbaszadegan

**Affiliations:** 1. Department of Microbiology, Damghan Branch, Islamic Azad University, Damghan, Iran; 2. Human Genetic Division, Immunology Research Center, Avicenna Research Institute, Mashhad University of Medical Sciences, Mashhad, Iran; 3. Department of Biology, Damghan Branch, Islamic Azad University, Damghan, Iran; 4. Cellular and Molecular Research Center, Sabzevar University of Medical Sciences, Sabzevar, Iran; 5. Department of Pathology, Imam Reza Hospital, Mashhad University of Medical Sciences, Mashhad, Iran; 6. Medical Genetics Research Center, Faculty of Medicine, Mashhad University of Medical Sciences, Mashhad, Iran

**Keywords:** Carcinogenesis, Esophageal squamous cell carcinoma, Germ cells, Testis

## Abstract

**Background:**

Cancer/Testis Antigens (CTAs) are a sub-group of tumor-associated antigens which are expressed normally in germ line cells and trophoblast, and aberrantly in a variety of malignancies. One of the most important CTAs is Developmental Pluripotency Associated-2(DPPA2) with unknown biological function. Considering the importance of *DPPA2* in developmental events and cancer, preparing a suitable platform to analyze *DPPA2* roles in the cells seems to be necessary.

**Methods:**

In this study, the coding sequence of *DPPA2* gene was amplified and cloned into the retroviral expression vector to produce recombinant retrovirus. The viral particles were transducted to Esophageal Squamous Cell Carcinoma (ESCC) cell line (KYSE-30 cells) and the stable transducted cells were confirmed for ectopic expression of *DPPA2* gene by real-time PCR.

**Results:**

According to the critical characteristics of retroviral expression system such as stable and long time expression of interested gene and also being safe due to deletion of retroviral pathogenic genes, this system was used to induce expression of *DPPA2* gene and a valuable platform to analyze its biological function was prepared. Transduction results clearly showed efficient overexpression of the gene in target cells in protein level due to high level of GFP expression.

**Conclusion:**

Such strategies can be used to produce high levels of desired protein in target cells as a therapeutic target. The produced recombinant cells may present a valuable platform to analyze the effect of *DPPA2* ectopic expression in target cells. Moreover, the introduction of its potential capacity into the mouse model to evaluate the tumorigenesis of these cancer cells *in vivo* leads to an understanding of the biological importance of *DPPA2* in tumorigenesis. In addition, our purified protein can be used in a mouse model to produce specific antibody developing a reliable detection of *DPPA2* existence in any biological fluid through ELISA system.

## Introduction

Epigenetic modifications such as CpG DNA methylation are widely reprogrammed on a genome during embryogenesis. The embryonic cells of blastocyst, Inner Cell Mass (ICM) and the Primordial Stem Cells (PSCs) are categorized as totipotent stem cells which can differentiate into all different types of individual cells^1–3^. Cancer cells share many similarities with germ line and embryonic cells, including deprogramming, invasive growth, proliferation, ability to self-renew and maintenance of the undifferentiated cell state. Therefore, it seems that embryonic active genes may be associated with these features of cancer cells. This hypothesis existed based on the identification of several embryo-cancer transcripts which are expressed in human embryos and absent in normal differentiated somatic cells, but re-expressed in tumor cells^4,5^. One of these transcripts is Developmental Pluripotency Associated-2 (DPPA2) which subsequently entered into the gene databases as Embryo-Cancer Sequence A (ECSA) and is also known as Cancer Testis Antigen100 (CTA100)^6,7^. Cancer Testis Antigens (CTAs) are one of the most promising categories of Tumor-Associated Antigens (TAAs) in cancer-therapy, and over 140 members of CTAs, globally accounting for about 70 families, have been identified^8–14^. The expression of CTAs normally restricted to germ cells in the testes, ovaries or trophoblasts^10,15,16^. CTAs are divided into two groups, the X-CTAs which are encoded by the X chromosome and represent about one-half of CT genes, and the non-X-CTAs which are distributed throughout the genome and are mostly single-copy genes. It has been estimated that 10% of the genes on the X chromosome belonged to CTAs^10,17,18^. The gene encoding *DPPA2* in human maps to chromosome 3q13 over 8 exons and encodes a protein consisting of 297 amino acids. The primary structure of this protein contains a Spliceosome-Associated Protein (SAP) motif and localizes to the nucleus. This gene is expressed in different types of malignancies including lymphoma and lung, ovarian, liver, and colon cancers^4,6^.

Few studies have focused on molecular epidemiology of *DPPA2* in cancer^19^, and the biological function of this protein is unclear. Our aim in this study was to produce a stable transducted cell line which constitutively express *DPPA2* gene, helping to understand well about the *DPPA2* biological role. A retroviral expression system was used in packaging cells to achieve high titers of recombinant virus particles. The results presented in this study demonstrate the feasibility of using this approach in expression of *DPPA2* and to survey the function of this protein in any target cells.

## Materials and Methods

### Gene analysis

The information of *DPPA2* gene was obtained from NCBI database. The main characteristics of the gene such as gene and mRNA lengths, the coding sequence, and the number of probable pseudogenes were analyzed.

### Primer design

After analyzing *DPPA2* gene in different databases and a survey for its pseudogenes, cloning primer set1 was designed using GeneRunner software version 3.05 (Hastings Software Inc., Hastings, NY, USA) and checked for probable hairpins, dimers, GC percentage and Tm.

### RNA extraction, cDNA synthesis

Having searched protein atlas database (http://www.proteinatlas.org), it was shown that *DPPA2* expressed cell lines. The selected cell lines were cultured, harvested, their total RNA were extracted using Trizol reagent (Invitrogen, Carlsbad, CA) and related cDNA was synthesized using oligo dT in first-strand cDNA synthesis kit (Fermentas, Vilnius, Lithuania) according to the manufacture’s protocols.

### Real time PCR

cDNAs were amplified on the Stratagene Mx-3000P real-time thermocycler (Stratagene, La Jolla, CA) with SYBR green mastermix (Invitrogen, Carlsbad, CA) containing ROX as a reference dye and real time primer set presented in table 1. All experiments were performed in duplicates.

**Table 1.** Real time primer set (1), Cloning primer set (2)

**Primer**	**Primer sequences**
**Forward primer 1**	5′-AGAAATACAATCCAGGTCATCTACTTC-3′
**Reverse primer 1**	5′-GCATATCTTGCCGTTGTTCAGG-3′
**Forward primer 2**	5′-TTTTGGATCCCAGGGTGTTGCT-3′
**Reverse primer 2**	5′-TTTTCTCGAGGTTGCTGCTACTTC-3′

### Amplification of *DPPA2* coding sequence

One microliter of cDNA (100 *ng* of total RNA) was used in PCR reaction with a final concentration of 1 *mmol/L* magnesium chloride, 0.5 *mmol/L* deoxynucleoside triphosphate (Fermentas, Vilnius, Lithuania), 0.2 units of Taq DNA polymerase (Fermentas, Vilnius, Lithuania), and 0/3 *μl* of each cloning primer set 1 in a final volume of 20 *μl*. The PCR condition was 95°*C* for 5 *min* followed by 35 cycles of 95°*C* for 30 *s*, 55°*C* for 30 *s* and 72°*C* for 45 *s*.

Cycling was terminated with a final extension step of 7 *min* at 72°*C*. PCR products were visualized on a 1% agarose/green viewer gel.

### TA cloning of amplified fragment

RT-PCR product was cut off from agarose gel and purified using DNA extraction kit (Invitek GmbH, Berlin, Germany), according to the manufacturer’s recommendation. The purified PCR product (5 to 10 *ng*) was cloned using TA cloning kit (Fermentas, Vilnius, Lithuania), according to the manual provided by the supplier and followed by blue/white selection. 15 colonies of transformed *Escherichia coli (E. coli) TOP10F’* were picked, cultured, and subjected to colony PCR. Plasmid extraction was done on confirmed recombinant colonies using plasmid extraction kit (GeNet Bio, Chungnam, Korea) and extracted plasmids were confirmed through colony PCR, double digestion with BamHI and XhoI restriction endonucleases and DNA sequencing.

### Characteristics of retroviral vector

The retroviral vector backbone used in this study, pRUF-IRES-GFP, is a Murine Leukemia Virus-Based Vector which utilizes a MLV long terminal repeat (LTR). This vector was kindly provided by Dr. Paul Moretti (Hanson Institute, SA, Australia, http://www.hansoninstitute.sa.gov.au/).

### Sub cloning of amplified fragment in retroviral vector

The pRUF plasmid was double digested with BamHI and XhoI and purified coding fragment of *DPPA2* gene was ligated into the cloning site of pRUF which is cleaved with the same enzymes. After transformation, 10 colonies of transformed *E. coli TOP10F’* were picked up, cultured, and subjected to plasmid extraction. The extracted plasmids were confirmed through colony PCR, double digestion with BamHI and XhoI enzymes and DNA sequencing.

### Cell lines and culture

GP293 and HEK293 (the Human Embryonic Kidney cells 293T) as packaging cell lines and KYSE-30 [Esophageal Squamous Cell Carcinoma (ESCC) cell line] as target cell line were cultured in DMEM and RPMI 1640, respectively, and both media were supplemented with 10% FBS (Gibco, NY, USA), 1% (*v/v*) penicillin, streptomycin and incubated in an atmosphere of 5% CO_2_ at 37°*C*.

### Retroviral vector production

High amount of pRUF-*DPPA2*, pVSV-G and pGP plasmids were produced in *E. coli* strain *TOP10F’* grown in LB medium supplemented with ampicillin (50 *mg/ml*). Plasmids were isolated and purified using Jetstar plasmid purification kit (Genomed, Bad Oeynhausen, Germany). Quality and quantity of the plasmids were verified by gel electrophoresis and UV-spectrophotometry. For transfection in 10 *cm* plate, 100 *ng* of each recombinant pRUF plasmid, plasmid encoding VSV-G protein and plasmid encoding Gag-Pol proteins were co-transfected into packaging cell lines separately using calcium phosphate method according to Tronolab protocol. Used solutions in this protocol included calcium phosphate: 2.5 *M* solution in ddH_2_O, sterile-filtered, 2× HEPES-Buffered Saline (HBS): (for 500 *ml*) 8 *g* NaCl, 0.38 *g* KCl, 0.1 *g* Na_2_HPO_4_, 5 *g* HEPES, 1 *g* glucose; pH=7.05, sterile-filtered, MEM C supplemented with 25 *mM* glucose. This protocol was adapted for 10 *cm* dishes but it can be adapted to smaller or bigger plates. The timing has also been optimized for our needs but it supports some flexibility. This protocol was done in 5 days. Day 1 (Plating); the day before transfection, HEK293T cells were suspended in 90% DMEM/10% FCS (*v/v*) and seeded into cell-culture dishes of 10 *cm* in diameter at a density of 2.5–3 *million cells/plate*. Day 2 (Transfection); transfection was carried out by the calcium phosphate method. For calcium phosphate precipitation (1 *ml*/90 *mm* culture dish), 20 *μg* of transfer vector (pRUF), 15 *μg* of packaging plasmid (pGP) and 6 *μg* of envelope plasmid (pVSV-G) were put into a sterile reaction tube in final volume of approximately 150–200 *μl*. Then 50 *μl* of 2.5 *M* calcium chloride solution was added to this plasmid solution. The solution was brought to a volume of 500 *μl* with autoclaved ddH_2_O. After transferring 500 *μl* of double-concentrated HBS solution to another reaction tube, the calcium chloride solution was added to the HBS solution very slowly (dropwise), and the solution was mixed. Incubation at room temperature for 1 *min* was followed by dropwise addition of the precipitate to the cells under *g*entle shaking of the plate. The plate was then incubated at 37°*C* in a humidified atmosphere of 95% air and 5% CO_2_. After 6–8 *hr* of transfection, the calcium phosphate precipitate-containing medium was removed and the cells were washed briefly with 5 *ml* of 90% DMEM/10% FCS (*v/v*), then 6 *ml* of fresh MEMC supplemented with 25 *mM* glucose were added and the plate was incubated at 37°*C* in a humidified atmosphere of 95% air and 5% CO_2_. Days 3, 4 and 5 (Collection of virus); transfected cells were examined under an inverted fluorescence microscope (Olympus IX-70). A narrow band of GFP filter set (exciter D480/20; emittor D520/20; Chroma, Brattleboro, VT) was used to detect the expression of the GFP in the cells. The virus-containing supernatant was harvested into a sterile tube, centrifuged (3000 *rpm*, 5 *min*, RT), filtered through a 0.45 *μm* mesh filter and stored at 4°*C*. Then the supernatant was ultracentrifuged in the SW28 rotor (Beckman Instruments, Fullerton, CA, USA) at 73000 *g* at 4°*C* for 2 *hr* to pellet the virus. After ultracentrifugation, the supernatant was aspirated and the pelleted virus was resuspended in a small volume of cultivation medium and stored in aliquots at −80°*C*. Transfection of 293GP cells was carried out using the same protocol as HEK-293 cells but just with *DPPA2*-pRUF and pVSV-G plasmids.

### Transduction of target cells by enriched recombinant viral particles

For transduction in a six-well plate, cells were seeded prior to the day for transduction. One *ml* of virus supernatant was mixed with 1 *ml* of fresh RPMI/10% FBS containing 1% pen/strep and added to wells. The cell culture medium was changed 5–10 *hr* after transduction. For detection of GFP expression, transduced cells were harvested 36–48 *hr* after transduction. Cells were examined under an inverted fluorescence microscope (Olympus IX-70, Tokyo, Japan). A narrow band of GFP filter set (exciter D480/20; emittor D520/20; Chroma, Brattleboro, VT) was used to detect the expression of the GFP in the cells.

### Titration of retroviral vector by quantitative PCR (qPCR)

KYSE-30 cells were transduced and the DNA was extracted using a genomic DNA extraction kit (Qiagen, Hilden, Germany). A fraction of this DNA was then analyzed for copy number of retroviral sequences using the Tronolab real-time PCR protocol. It measured the number of retroviral DNA copies integrated in the target cell genome. The ultimate test of the functionality of the vector was in cells supporting the activity of the promoter driving the transgene. Titration of retroviral particles was performed according to Tronolab protocol^20^. Firstly, a mix (containing everything but the sample DNA) for the number of wells needed for the qPCR analysis, including all samples and standards in duplicates according to the following recipe (9 *μl* per well) was prepared: 10 *μl* SYBR green PCR Master Mix (Fermentas, Lithuania) and 1 *μl* forward/reverse mix primer (10 *pM*). Then, 2 *μl* of sample DNA to each of the appropriate wells was added. The amplification cycles used were: 1 cycle: 10 *min* 95°*C*, then 40 cycles: 15 *s* 95°*C*, 30 *s* 60°*C* and 20 *s* 72°*C*. pAlb (available from Addgene, http://www.addgene.org) was a pRRL vector in which the target sequence of the albumin primers used for normalization has been cloned. This plasmid allows performing a standard curve. Gag oligos were used for amplification of pRUF vector sequence and were specific for the 5′ end of the *gag* gene. This sequence was present in pRUF vector, as it was part of the extended packaging signal. Alb oligos were used to normalize the amount of genomic DNA and were specific for the human albumin gene (Table 2). Also cells were examined under an inverted fluorescence microscope (Olympus IX-70). A narrow band of GFP filter set (exciter D480/20; emittor D520/20; Chroma, Brattleboro, VT) was used to detect the expression of the GFP in the cells.

**Table 2.** Primers for retrovectors’ titration by qPCR

**Sequence detected**	**Primer name**	**Primer sequence**
**Gag**		
	Gag forward	GGAGCTAGAACGATTCGCAGTTA
	Gag reverse	GGTGTAGCTGTCCCAGTATTTGTC
**Albumin**		
	Alb forward	GCTGTCATCTCTTGTGGGCTGT
	Alb reverse	ACTCATGGGAGCTGCTGGTTC

## Results

### Gene analysis and primer design

*DPPA2* gene was analyzed using NCBI database. The mRNA length of the gene is 1393 *bp* containing 9 exons. The coding region on mRNA is from 248 to 1144 *bp* which encodes 298aa. A pseudogene was found for *DPPA2* coding sequence which is important in primer design. Alignment between the gene and its pseudogene was done using CLC work bench software version 5.6 (CLC bio, Aarhus, Denmark), and the percentage of homology was 92%. Since the length of pseudogene was 248 *bp* shorter than the *DPPA2* mRNA at 5′ end and due to the similar sequences of primary regions, forward primer designed 4 nucleotides before the ATG initiation codon. Having analyzed the gene sequence, a cloning primer set presented in table 1 was designed. By substitution of 2 and 3 nucleotides, restriction sites were induced for BamHI (GGATCC) and XhoI (CTCGAG) in forward and reverse primers, respectively (Table 1).

### RNA extraction, cDNA synthesis

Five cell lines including SKOV3, NCCIT, HEK293, Hela and HT that expressed *DPPA2* gene were found using protein atlas site. Full length complementary DNA (cDNA) of *DPPA2* coding sequence was synthesized from the total RNA extracted from SKOV3, NCCIT, HEK293, Hela, HT cell lines. cDNA of SKOV3 cell line was also synthesized by downstream primer.

### Real time PCR

The expression level of *DPPA2* in these cell lines was determined using real time PCR. The results revealed that SKOV3 cell line has higher expression level than others.

### Reverse transcription-polymerase chain reaction (RT-PCR) assay

**Figure 1.**
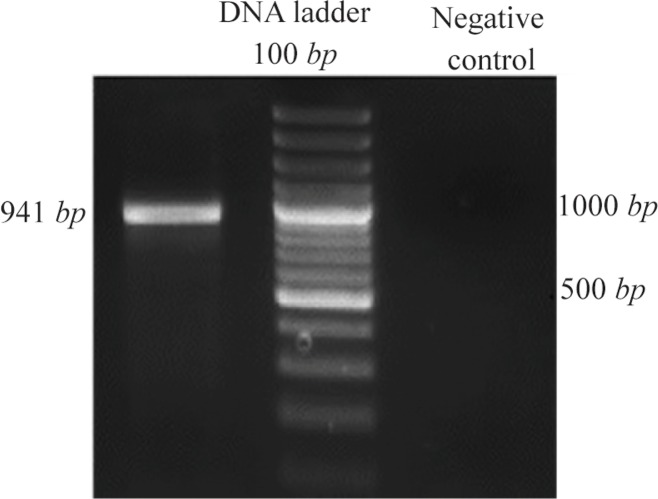
PCR amplification of *DPPA2*. PCR product was 941 *bp* long as expected when visualized on agarose gel.

cDNA was amplified with gene specific primers by standardized PCR conditions. Resulting fragment of *DPPA2* was 941 *bp* in size as expected when visualized on agarose gel (Figure 1).

### TA cloning

The amplified product was used for ligation in pTZ57R/T cloning vector. The basis for ligation of any PCR product in this vector is the presence of over hanged adenines at both 3′ and 5′ ends of PCR product which binds complementary with thymine present at MCS of the linearized vector during ligation process.

### Transformation of *E. coli* with pTZ57R/T *DPPA2* vector

Competent cells of *E. coli* strain, *TOP10F’,* were transformed with pTZ57R/T*DPPA2* vector using cacl2 method. Insertion of *DPPA2* cDNA in multiple cloning sites of pTZ57R/T caused in activation of *Lac Z* gene hence white colonies were produced on selection with X-Gal/IPTG containing media plates. Single transformed white colonies were further used for colony PCR and plasmid extraction. The size of pTZ57R/T is 2886 *bp* which was increased to 3827 *bp* after insertion of *DPPA2* fragment and visualized by agarose gel electrophoresis.

### Confirmation of *DPPA2* through colony PCR, restriction digestion and sequencing

**Figure 2.**
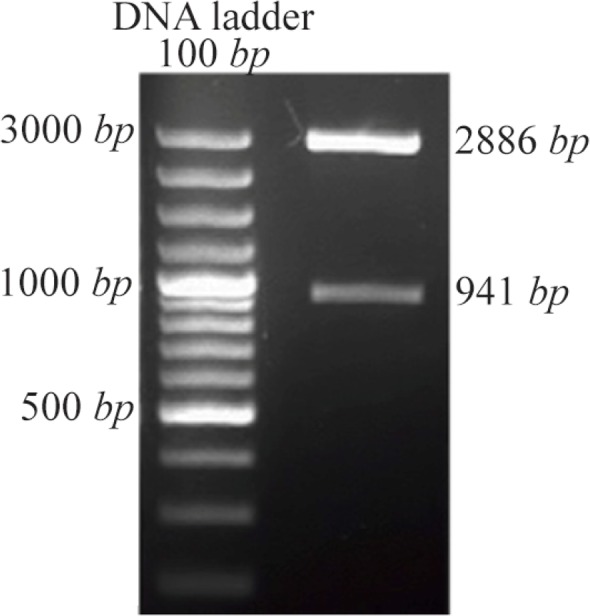
Recombinant pTZ57R/TDPPA2 digested with BamHI and XhoI. Digestion of recombinant pTZ57R/TDPPA2 gives 2 bands that correspond to pTZ57R/T vector (2886 *bp*) and *DPPA2* gene (941 *bp*).

After confirming through colony PCR, the recombinant plasmid was extracted and digested with BamHI and XhoI resulting in release of about 941 *bp* fragment which was separated on 1% agarose gel (Figure 2). Finally, the sequence of PCR product in pTZ57R/TDPPA2 vector was confirmed by sequencing.

### Sub cloning of *DPPA2* gene in pRUF expression vector

**Figure 3.**
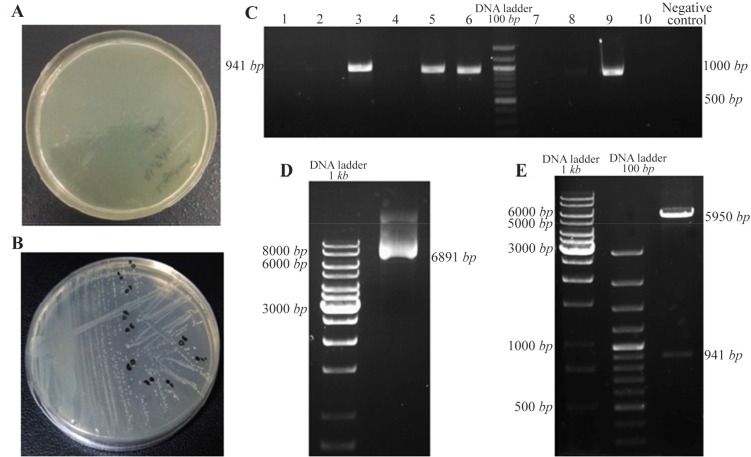
Sub cloning of *DPPA2* gene in pRUF expression vector, A. Negative control plate; B. Sub cloning plate of *DPPA2* gene; C. Colony PCR for confirming recombinant colonies, D. The size of pRUF vector was increased to 6891 *bp* after insertion of *DPPA2* gene, E. Digestion of recombinant pRUF-*DPPA2* plasmid by BamHI and XhoI gives 2 bands that correspond to pRUF expression vector (5950 *bp*) and *DPPA2* gene (941 *bp*).

Competent cells of *E. coli* strain, *TOP10F,’* were transformed with pRUF-*DPPA2* vector.10 colonies of transformed *E. coli* were chosen and confirmed through colony PCR and double digested with BamHI and XhoI enzymes. The size of pRUF is 5950 *bp* which was increased to 6891 *bp* after insertion of *DPPA2* cDNA and visualized by agarose gel electrophoresis. After extraction of pRUF-*DPPA2* vector, this recombinant plasmid was digested with BamHI and XhoI resulting in release of about 941 *bp* fragment which was separated on 1% agarose gel to confirm the size (Figure 3).

### Retroviral vector production

**Figure 4.**
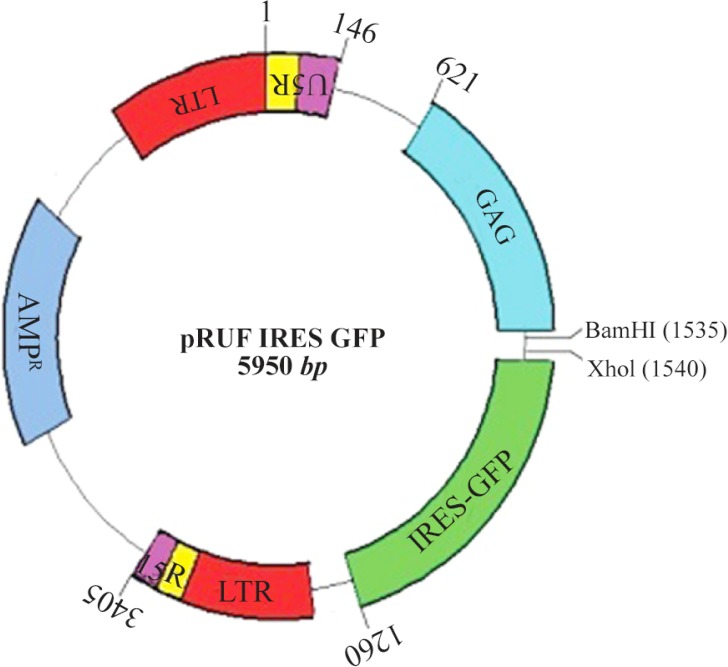
pRUF Retroviral Vector Map. This image was kindly provided by Paul Moretti, Hanson Institute, SA, Australia, http://www.hansoninstitute.sa.gov.au/

The pRUF plasmid is an expression vector containing *GFP* gene as a reporter gene, Internal Ribosome Entry Site (IRES), ampicillin resistant gene and restriction site for BamHI and XhoI enzymes (Figure 4).

**Figure 5A–C.**
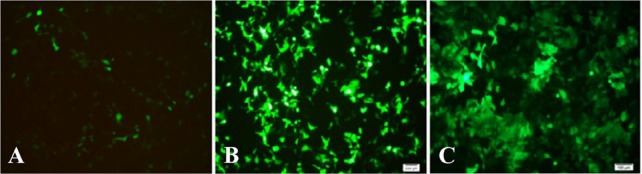
GFP expression in target cells 48 *hr* after transfection (×100) that were representative fluorescent photographs of HEK293T and GP293, respectively, under fluorescent microscope. 6C was representative fluorescent photograph of target cell (KYSE-30) that was transducted by enriched recombinant viral particles.

**Figure 6.**
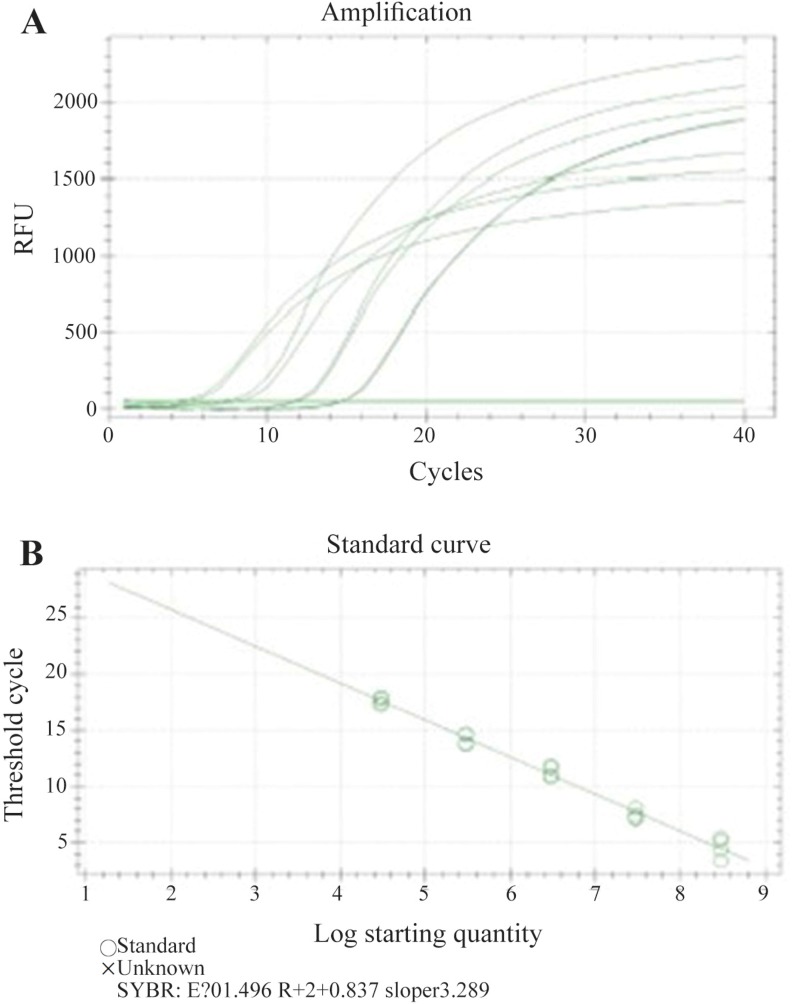
Titer estimation of retroviral vector using real-time RT-PCR. Serial dilutions of pAlb vector were prepared. A) Amplification plot of samples with each dilution was represented in order from left to right on the graph. B) Standard curve generated from amplification plot is shown in A. Each sample was performed in duplicate and is represented as a dot. Overlapping dots are present in most of the dilutions illustrating the tight correlation within each dilution. Correlation coefficient for Alb and Gag was 103.3 and 99.5%, respectively.

Recombinant pRUF-IRES-GFP was transiently co-transfected along with the plasmids encoding VSV-G and Gag-Pol proteins into the HEK293 packaging cells. Cells were examined under an inverted fluorescence microscope (Olympus IX-70, Tokyo, Japan). A narrow band of GFP filter set (exciter D480/20; emittor D520/20; Chroma, Brattleboro, VT) was used to detect the expression of the GFP in the cells. The efficiency of transfection was about 10%. Recombinant pRUF with VSV-G plasmid were cotransfected into 293GP packaging cells which express gag and pol genes. The efficiency of transfection into 293GP was about 70% that was higher than HEK293 retroviral packaging cells (Figures 5A and 5B). These percentages were determined by measurement of GFP expression by flow-cytometry analysis (data not shown). Viral supernatants were collected and used to infect KYSE-30 cells (Figure 5C). After transduction, viral titers were determined by quantitative PCR (qPCR), and virus stocks containing 10^5^*TU/ml* were obtained (Figure 6).

## Discussion

Deprogramming or removal of the epigenetic information returns the cell to the undifferentiated stem cell state^21–25^. Embryonic genes which are active in pluripotent embryonic stem cells may be associated with similar properties presented in cancer cells involving deprogramming, unlimited proliferation, maintenance of the undifferentiated cell state, invasiveness as well as the ability to self-renew. Therefore, cancer cells may resemble stem cells through common functions and signals to initiate and progress deprogramming^4,5^. Many cancers are associated with reactivation of germ cell and embryonic genes, e.g. the so-called *Cancer/Testis (CT)* genes or *CG* genes^14,15,26,27^. ECSA/*DPPA2*, also known as CT100, is a human embryonic antigen that is predominantly expressed in NSCLC (Non-small cell lung carcinoma) and also in other malignancies including melanoma, lymphoma, and in lung, liver and colorectal cancers^6,28^. This may confirm involvement of embryonic stem cell properties in the cancer cells or/and cancer stem cell phenotype^28^. Although the biological functions of this gene are not clear, ectopic expression of this transcription factor can help to understand the functional activity of this gene in the proliferation and maintenance of stem cell activity of cancer cells.

Up to now, several cell-targeting strategies have been developed to obtain the efficient mechanism for delivery of an interested gene to a particular target cell and operating its ectopic expression *in vitro*. Nucleic acids introduction into the target cells may be a medical purpose, and currently, different gene therapy studies are being developed^29,30^. Since efficient expression of gene of interest in target cell is an essential step, integration of the gene into the host genome can lead to a long-term gene expression^31^. Although several viral vectors have been developed for this reason, retroviral vectors may present promising qualities including large packaging size, long-term expression, capacity for cell targeting and scalable production^32,33^.

pRUF expression vector, as a retroviral vector, is a Murine Leukemia Virus-Based Vector. Retroviral vectors are needed for packaging cells such as HEK293 or GP293. In this study, pRUF expression vector along with pVSV-G and pGP plasmids were cotransfected to packaging cells. The results presented here indicate that the efficiency of transfection to HEK293 is lower than GP293 cell line. GP293 is a packaging cell that stably expresses Gag-Pol protein, so efficiency of transfection to GP293 is higher than HEK293. Several groups have recently reported the generation of high-titer retroviral particles using transient transfection systems. pRUF contains an extended packaging signal, which is believed to be important for generating high-titer viral preparations. To evaluate and monitor the gene transfer efficiency of these retroviral vectors, a *GFP* gene in this retroviral vector existed that exhibited fluorescence and was easily detected by fluorescence microscopy. pRUF is an IRES-GFP vector and *GFP* works as a reporter gene for monitoring and evaluation of viral transduction efficiency. By this retroviral vector system, there is no need to do western blotting and IHC assays for confirming the expression of interested gene.

In this research, *DPPA2* gene was amplified and isolated by RT-PCR and the efficiency of cloning was increased by using TA cloning. The universal TA cloning method relies on the supposition that all DNA fragments can be easily converted to double stranded DNA with over hanged adenines at both 3′ ends, and thus the T-vector becomes a ready-for-ligation universal cloning vector.

Our transduction results clearly showed efficient over expression of the gene in target cells in protein level due to high level of GFP expression. Such strategy can be used to produce high level of desired protein in target cell as a therapeutic target. Interestingly, different cellular and molecular biology approaches (such as real-time PCR for gene expression pattern profiling, and proliferation and migration assay for analysis of cell behavior), can be developed to analyze the effect of protein ectopic expression in target cells. Furthermore, the produced cells with ectopic expression of target gene can be introduced to the mouse model to evaluate the tumorigenesis of these cancer cells *in vivo*, leading to an understanding of the biological importance of *DPPA2* in tumorigenesis. In addition, purified protein can be used in a mouse model to produce specific antibody, developing a reliable detection of *DPPA2* existence in any biological fluid through ELISA system.

## Conclusion

In summary, *DPPA2* gene was successfully sub cloned and expressed in pRUF expression vector, and by producing a recombinant retrovirus, *DPPA2* gene was transducted and expressed in KYSE-30 cell as a target cell. Since the function of *DPPA2* is not clear, it can be used for understanding the biological function of this cancer testis antigen. Also, the recombinant *DPPA2* protein that was produced by this recombinant retrovirus can be used in production of recombinant vaccines.
